# Supplementary material for: Engineered clearing agents for the selective depletion of antigen-specific antibodies
Source: Nat Commun. 2017 May 31;8:15314. doi: 10.1038/ncomms15314 (PMC5460014; doi:10.1038/ncomms15314)
Supplement: Supplementary Information — Supplementary Figures and Supplementary Table [file ncomms15314-s1.pdf]

Supplementary Figure 1

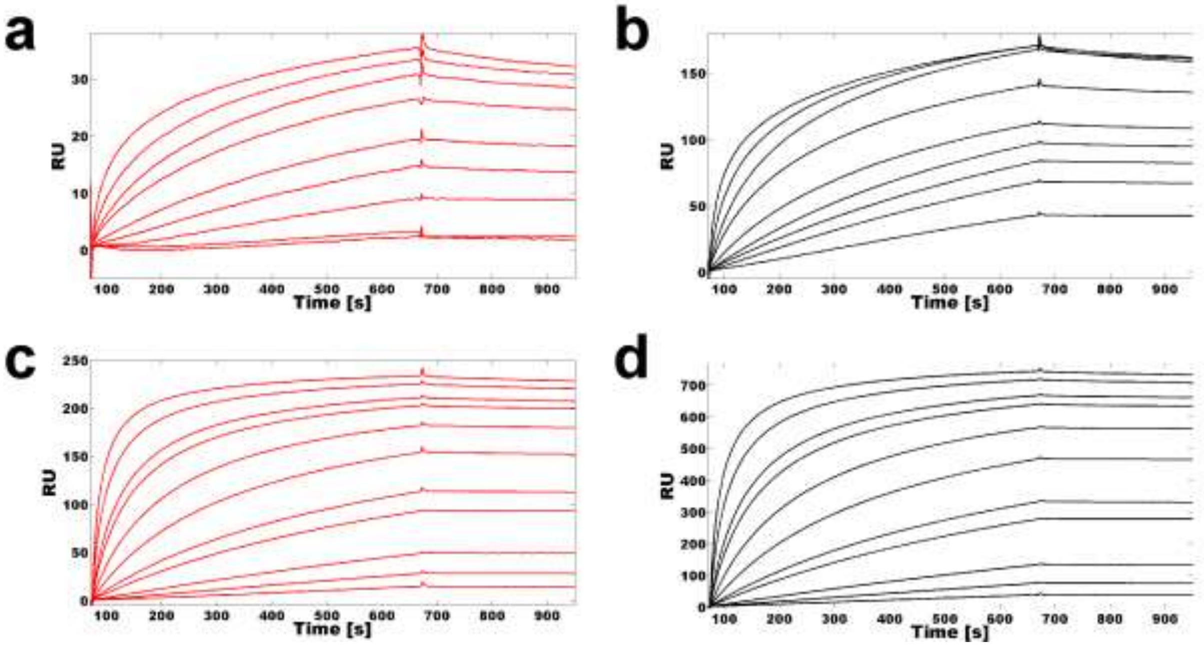

Supplementary Figure 1. Surface plasmon resonance analyses to determine the equilibrium dissociation constants of the interactions of Seldegs with their corresponding antigen-specific antibodies. Sensorgrams for the interactions of different concentrations of 8-18C5 with MOG-Seldeg (**a**) or MOG-WT (**b**) and TZB with HER2-Seldeg (**c**) or HER2-WT (**d**) are shown. Antibodies were injected over immobilized MOG-Seldeg, MOG-WT, HER2-Seldeg or HER2-WT at concentrations ranging from 1.6-500 nM (**a, b**) or 0.8-500 nM (**c,d**) at a flow rate of 10 μl/min. Each analyte concentration was injected at least twice during the equilibrium run, and representative individual traces are presented. Data shown are representative of two independent experiments.

Supplementary Figure 2

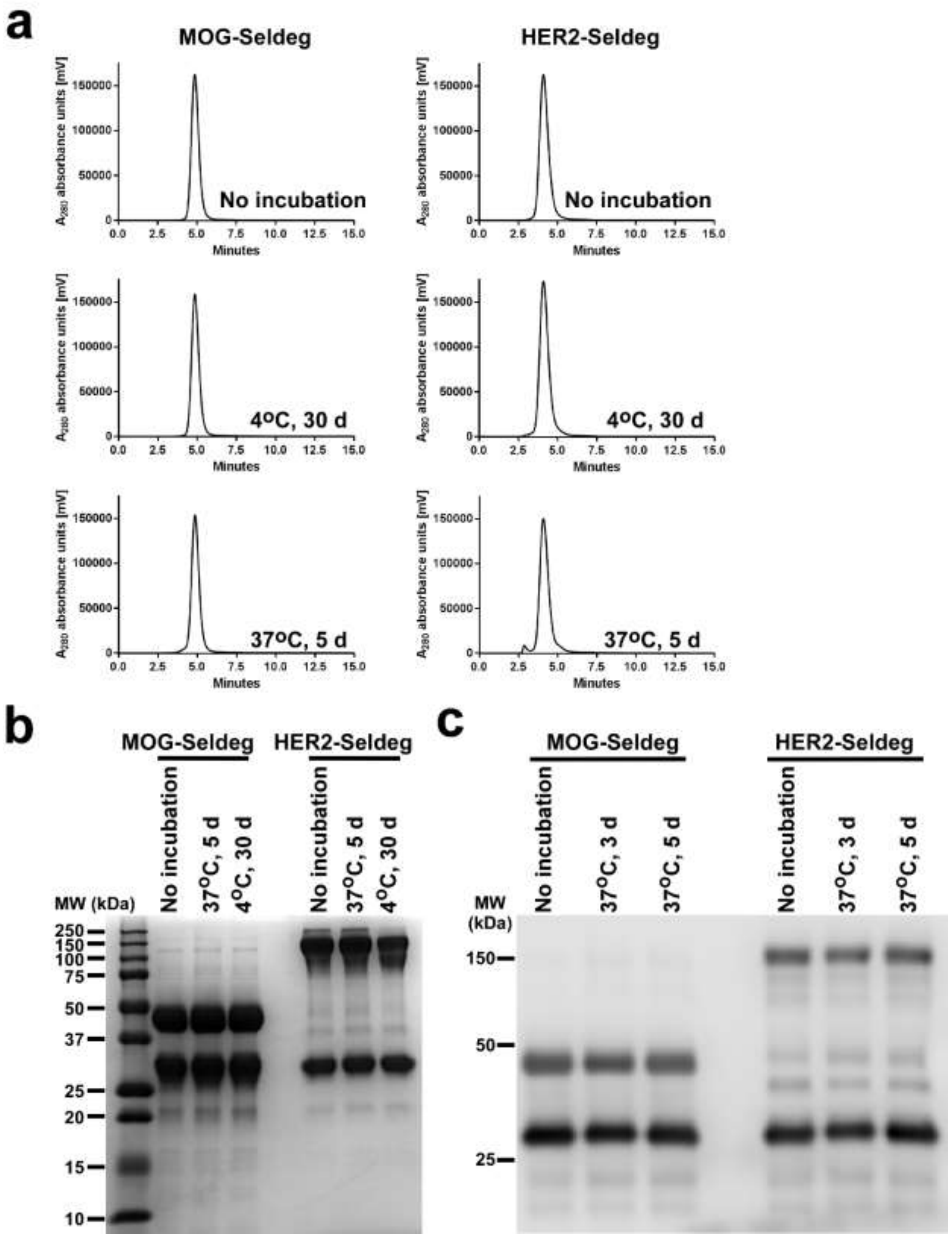

Supplementary Figure 2. Analyses of the stability of Seldegs following storage at 4°C or incubation at 37°C. **a**, Seldegs were stored in PBS at 37°C for 5 days (5 d) or 4°C for 30 days (30 d) followed by analyses on a Superdex 200 5/150 GL column. **b**, SDS-PAGE analysis of Seldegs following storage in PBS at 37°C for 5 days or 4°C for 30 days. **c**, Seldegs were incubated at 37°C for 3 or 5 days in IgG-depleted human serum at a concentration of 100 nM. Incubated Seldegs were immunoprecipitated and analyzed by immunoblotting using goat anti-human (H+L) antibody conjugated with HRP. For **b** and **c**, sizes of molecular weight standards are shown in kDa on the left margins. For **a**-**c**, 'no incubation' indicates that recombinant proteins were stored frozen and thawed immediately before analysis. Data shown are representative of two independent experiments.

Supplementary Figure 3

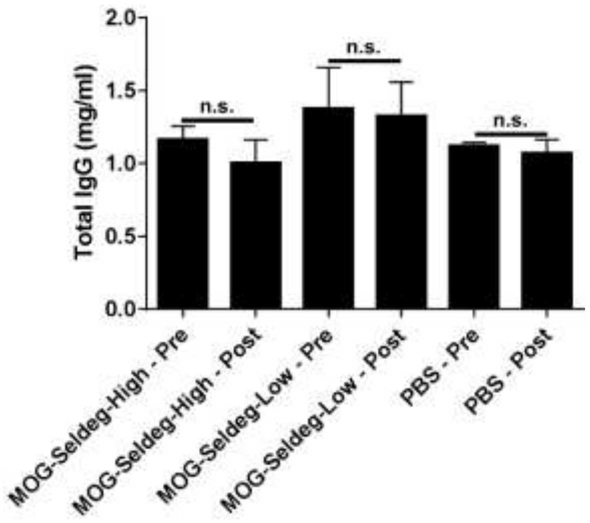

Supplementary Figure 3. MOG-Seldeg delivery does not change the total serum IgG levels in mice. Mice (n = 6 per group) were bled before ('pre') and 48 hours following ('post') the delivery of 31 μg (low dose) or 125 μg (high dose) MOG-Seldeg. Endogenous serum IgG levels were determined for triplicate samples for each mouse by ELISA. Error bars indicate standard deviations. Statistically significant differences for IgG levels before and following Seldeg (or control PBS) treatment were analyzed using Student's t-test. n.s., no significant difference ($p > 0.05$). Data shown are representative of two independent experiments.

Supplementary Figure 4

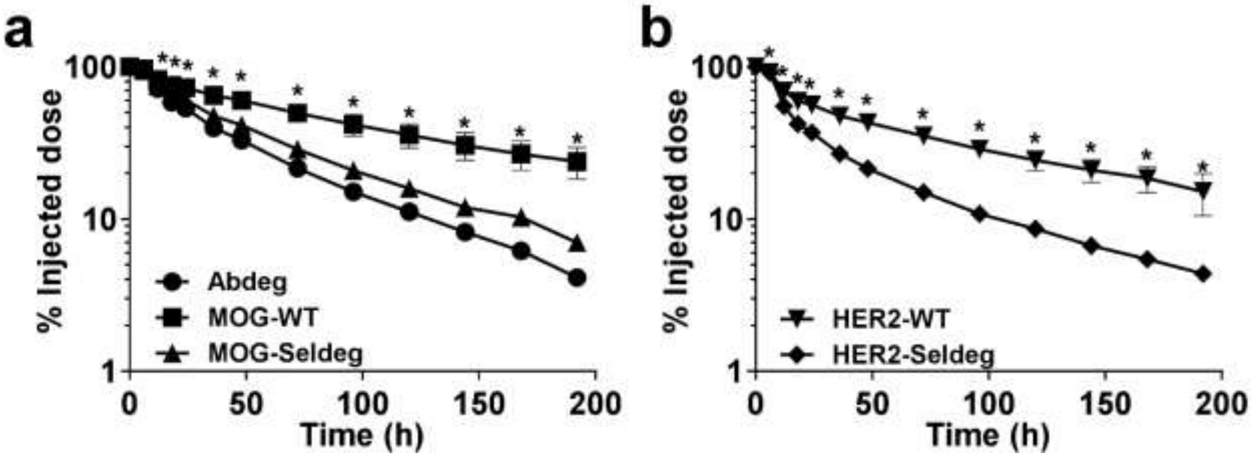

Supplementary Figure 4. Seldegs are cleared more rapidly in transgenic C57BL/6 mice expressing huFcγRs than their corresponding WT fusion proteins. Radiolabeled (^{125}I) MOG-WT/Seldeg, HER2-WT/Seldeg and Abdeg were delivered and whole body counts obtained at the indicated time points. Whole body levels immediately following injection were taken as 100%. Error bars indicate standard deviations. Statistically significant differences are indicated for MOG-WT vs. MOG-Seldeg (**a**) or HER2-WT vs. HER2-Seldeg (**b**) by * ($p < 0.05$, two-way ANOVA with Tukey's multiple comparisons; $n = 5$ mice/group). Data shown are representative of two independent experiments.

Supplementary Figure 5

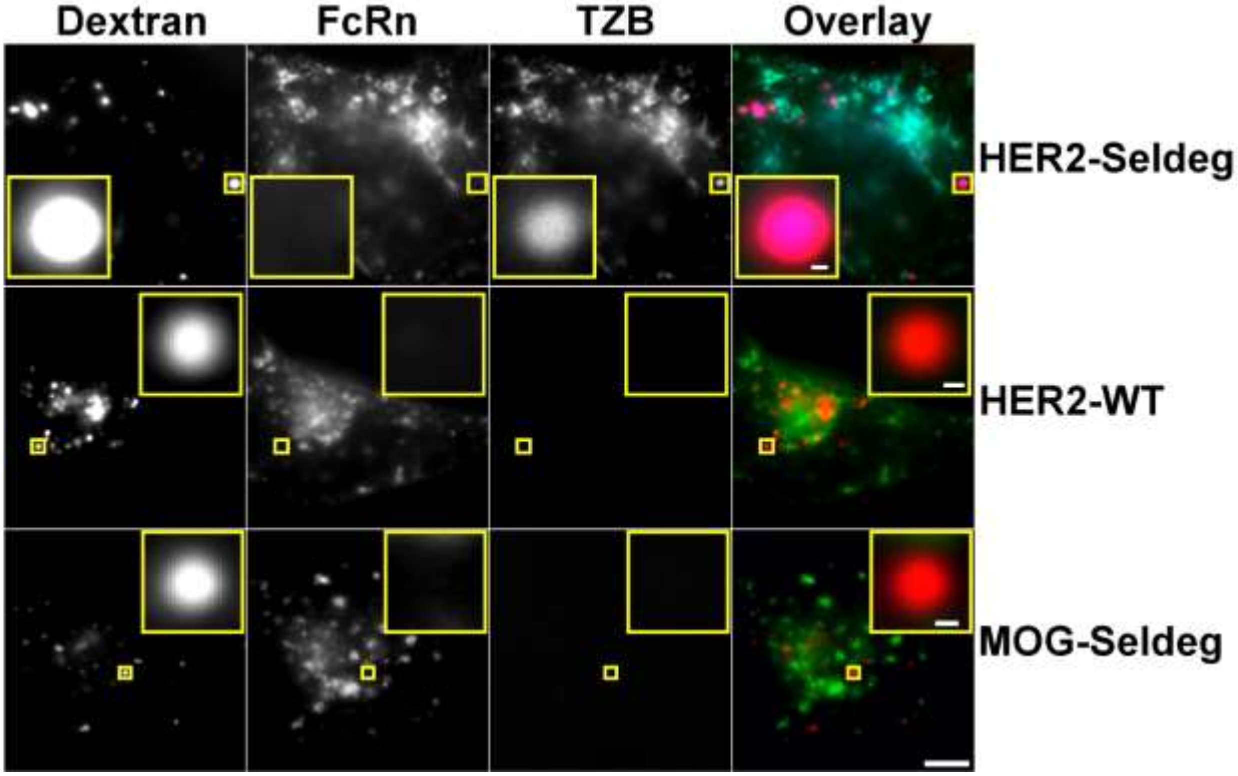

Supplementary Figure 5. The accumulation of antigen-specific antibodies in the lysosomes of FcRn-GFP expressing human endothelial (HMEC-1) cells is dependent on antigen binding. HMEC-1 cells were pre-pulsed with Alexa 555-labeled Dextran for 2 hours, washed and pulsed with 100 nM Alexa 647-labeled TZB (HER2-specific) in complex with 400 nM HER2-Seldeg, HER2-WT, or MOG-Seldeg for 30 minutes followed by an 8 hour chase. Cells were washed, fixed and imaged following the chase period, and images for a representative cell from multiple cells analyzed are presented. Representative lysosomes in the insets are cropped and expanded. For the overlay, GFP, Alexa 555 and Alexa 647 are pseudocolored green, red and blue, respectively.

Bars = 5 μm, and for insets, bars = 0.25 μm. Data shown are representative of at least two independent experiments.

Supplementary Table 1

Equilibrium dissociation constants (K_D, nM) of the interactions between recombinant Fc fusion proteins and antibodies[1]

Fc-fusion protein	HER2-specific (TZB)	MOG-specific (8-18C5)
**MOG-WT**	n.b.[2]	16.5
**MOG-Seldeg**	n.b.[2]	39.5
**HER2-WT**	15.8	n.b.[2]
**HER2-Seldeg**	12.9	n.b.[2]

[1]Corresponding sensorgrams are shown in Supplementary Figure 1; [2]n.b., no detectable binding.
